# 2021 EULAR recommendations for the implementation of self-management strategies in patients with inflammatory arthritis

**DOI:** 10.1136/annrheumdis-2021-220249

**Published:** 2021-05-07

**Authors:** Elena Nikiphorou, Eduardo José Ferreira Santos, Andrea Marques, Peter Böhm, Johannes WJ Bijlsma, Claire Immediato Daien, Bente Appel Esbensen, Ricardo J O Ferreira, George E Fragoulis, Pat Holmes, Hayley McBain, George S Metsios, Rikke Helene Moe, Tanja A Stamm, Annette de Thurah, Condruta Zabalan, Loreto Carmona, Ailsa Bosworth

**Affiliations:** 1 Rheumatology Department, King's College Hospital, London, UK; 2 Centre for Rheumatic Diseases, King’s College London, London, UK; 3 Rheumatology, Centro Hospitalar e Universitário de Coimbra EPE, Coimbra, Portugal; 4 Health Sciences Research Unit Nursing, Higher School of Nursing of Coimbra, Coimbra, Portugal; 5 German League against Rheumatism, Bonn, Germany; 6 Department of Rheumatology & Clinical Immunology, University Medical Center Utrecht, Utrecht, The Netherlands; 7 Lapeyronie Hospital, CHU Montpellier, and Inserm U1046, CNRS UMR 9214, Montpellier University, Montpellier, France; 8 Copenhagen Center for Arthritis Research (COPECARE), Center for Rheumatology and Spine Diseases, Centre of Head and Orthopaedics, Rigshospitalet, Denmark; 9 Department of Clinical Medicine, Faculty of Health and Medical Sciences, University of Copenhagen, Copenhagen, Denmark; 10 First Department of Internal Medicine, Propaedeutic Clinic, Athens, Greece; 11 National Rheumatoid Arthritis Society, Maidenhead, UK; 12 School of Health Sciences, City, University of London, London, UK; 13 Faculty of Education, Health and Wellbeing, University of Wolverhampton, Walsall, UK; 14 Department of Nutrition and Dietetics, University of Thessaly, Trikala, Thessaly, Greece; 15 Division of Rheumatology and Research, Diakonhjemmet Hospital, Oslo, Norway; 16 Section for Outcomes Research, Center for Medical Statistics, Informatics, and Intelligent Systems, Medical University of Vienna, Vienna, Austria; 17 Department of Rheumatology, Aarhus University Hospital, Aarhus, Denmark; 18 Departent of Clinical Medicine, Aarhus University, Aarhus, Denmark; 19 Romanian League against Rheumatism, Bucharest, Romania; 20 Instituto de Salud Musculoesquelética, Madrid, Spain; 21 National Rheumatoid Arthritis Society, Littlewick Green, UK

**Keywords:** arthritis, rheumatoid, outcome assessment, health care, psoriatic, spondylitis, ankylosing

## Abstract

**Background:**

An important but often insufficient aspect of care in people with inflammatory arthritis (IA) is empowering patients to acquire a good understanding of their disease and building their ability to deal effectively with the practical, physical and psychological impacts of it. Self-management skills can be helpful in this regard.

**Objectives:**

To develop recommendations for the implementation of self-management strategies in IA.

**Methods:**

A multidisciplinary taskforce of 18 members from 11 European countries was convened. A systematic review and other supportive information (survey of healthcare professionals (HCPs) and patient organisations) were used to formulate the recommendations.

**Results:**

Three overarching principles and nine recommendations were formulated. These focused on empowering patients to become active partners of the team and to take a more proactive role. The importance of patient education and key self-management interventions such as problem solving, goal setting and cognitive behavioural therapy were highlighted. Role of patient organisations and HCPs in promoting and signposting patients to available resources has been highlighted through the promotion of physical activity, lifestyle advice, support with mental health aspects and ability to remain at work. Digital healthcare is essential in supporting and optimising self-management and the HCPs need to be aware of available resources to signpost patients.

**Conclusion:**

These recommendations support the inclusion of self-management advice and resources in the routine management of people with IA and aim to empower and support patients and encourage a more holistic, patient-centred approach to care which could result in improved patient experience of care and outcomes.

**Key messages**

**What is already known on this subject?**

- The ability to self-manage in inflammatory arthritis (IA) represents an essential component of care that goes beyond drug therapy and which supports the patient in managing the practical, physical and psychological impacts of disease.
- Self-management is a multicomponent complex intervention that represents an unmet need in the care of people with IA.

**What does this study add?**

- These recommendations, based on evidence and expert opinion, confirm the beneficial effects of different components of self-management and provide guidance on embedding self-management interventions into the routine clinical care of people with IA.
- This work highlights the value of patient organisations in providing support and structured guidance for people with IA and the need to demonstrate and document the effectiveness of specific self-management interventions.

**How might this impact on clinical practice?**

- Adherence to these recommendations will lead to improved patient care and outcomes in people living with IA and will encourage a more active patient role in the management of disease.

## Introduction

In people living with inflammatory arthritis (IA), as well as other rheumatic and musculoskeletal diseases (RMDs) and chronic conditions, an important aspect of care is the ability to understand the disease and deal with the practical, physical and psychological impacts that come along with it.^1 2^ This extends beyond drug therapy and places emphasis on the ability to self-manage as an essential component of care.^3^ Comorbidities including cardiovascular disease and common mental health conditions represent important, yet often poorly addressed aspects of IA despite their impact on disease outcomes.^4 5^ Addressing physical as well as psychological comorbidities is therefore crucial and more likely to be achieved if more holistic approaches to patient care are adopted, including, for example, signposting, where appropriate, to other members of the multidisciplinary team (MDT).^6^ These members include, aside from rheumatologists, nurses, physiotherapists, occupational therapists, podiatrists, psychologists, nutritionists and any other healthcare professionals (HCPs) involved in the care of patients with IA.^2^ All these important aspects of disease which can place a high burden on the individual and their immediate family necessitate the incorporation of supported self-management in the routine clinical care of people living with IA. For self-management to be effective however, it is imperative that HCPs (for the purposes of this work, reference to HCPs includes rheumatologists as well as allied health professionals) are given adequate guidance and professional training. This has a significant positive impact on their engagement in clinical self-management support and patient centredness, as well as on their overall confidence to support self-management.^7^ Patient organisations also play a role in the provision of supported self-management resources. Acknowledging all these important aspects of care, a taskforce supported by the European Alliance of Associations for Rheumatology (EULAR) was convened to embed recommendations alongside the standard medical care of IA that encourage supported patient self-management and concordance with treatment.

The overarching aim of the taskforce was to formulate recommendations for the implementation of self-management strategies in patients with IA, including but not limited to rheumatoid arthritis, psoriatic arthritis and axial spondyloarthritis. The target audience was HCPs including all members of the MDT and patients. There were three key objectives: (1) to develop EULAR recommendations for the implementation of effective self-management strategies facilitated by HCPs in IA concurrently with and complimentary to the delivery of standard medical care, (2) to enable all members of the rheumatology MDT to be able to provide and signpost a continuous and appropriate measure of support to enable better self-management of patient with IA and (3) to improve the patient’s ‘journey’ and experience during their care, disease outcomes and quality of life.

## Methods

The 2014 updated EULAR standardised operating procedures were followed throughout the execution of this work.^8^ Following approval by the EULAR Executive Committee, the convenors (AB, EN) and methodologist (LC) led a taskforce of 18 members from across 11 European countries. Taskforce members came with a background and expertise in rheumatology, nursing, occupational therapy, psychology, self-management, exercise physiology and physiotherapy. The taskforce also included patient representatives with lived experience of IA from People with Arthritis/Rheumatism across Europe. Expert discussions took place primarily through two taskforce meetings, the first, face-to-face and the second, via a virtual online platform.

In preparation for the first meeting, an initial scoping review and a survey (available on request) were undertaken to explore, respectively, effective interventions in IA and self-management resources in RMDs across Europe. During the first meeting, the scope of this work, definitions for self-management and overarching principles (OAPs) were discussed. Furthermore, unmet need and key clinically relevant questions were identified in relation to self-management in IA and sources of best practice examples explored.

In preparation for the second meeting and, as guided by the first meeting, clinical questions were converted by the steering group (AB, EN, LC, AM, EJFS) into epidemiological questions that were addressed through systematic literature review (SLR) (under submission) undertaken by the taskforce fellows (AM, EJFS). The aim of the SLR was to identify the best evidence for the implementation of self-management interventions in IA and to describe individual components and effects. The review was conducted according to the Cochrane Handbook^9^ and reported in accordance with the Preferred Reporting Items for Systematic Reviews and Meta-Analyses guidelines.^10^ Patient organisations affiliated with EULAR and HCPs across Europe were also approached via direct email communication requesting information and experience/feedback on examples of self-management resources in IA, to supplement the information retrieved from the SLR.

At the second meeting, the taskforce members formulated the OAPs and recommendations based on evidence from the SLR, survey, email communication with patient organisations/HCPs and best practice examples, guided by their expert opinion and through a process of discussion and voting. Consensus was accepted in the first round if >75% of the members voted in favour of keeping it in. In the second and third rounds, after refinements, the level of agreement (LoA) was voted on a 0 to 10 scale (0=‘do not agree at all’ to 10=‘fully agree’) anonymously. The second round was voted through Zoom polls during the second meeting and the third round through SurveyMonkey, afterwards. The mean and SD of the LoA was presented along with the percentage of taskforce members with an agreement ≥8. An indication of the level of evidence (LoE) based on the evidence retrieved from the SLR was discussed for each of the formulated recommendations, to facilitate discussions. At the meeting, the LoE and strength of recommendation were assigned for each of the final drafted recommendations using the standards of the Oxford Centre for Evidence Based Medicine.^11^

Finally, a research agenda was formulated based on discussions around identified unmet need and gaps in evidence.

## Results

The taskforce discussed existing definitions for self-management and reached consensus on three OAPs and nine recommendations (table 1), guided by the results of the SLR, the surveys to patient organisations and HCPs relating to self-management resources, across EULAR countries (online supplemental file) and best practice examples (can be provided on request). In total, 12 patient organisations were approached of which 9 responded, representing eight different countries. A total of 13 HCPs were approached and 100% replied from 13 different countries.

10.1136/annrheumdis-2021-220249.supp1 Supplementary data

**Table 1:** EULAR overarching principles (OAPs) and recommendations for the implementation of self-management strategies in patients with inflammatory arthritis (IA)

	LoE (1–5)	SoR (A–D)	Level of agreement (0–10)	
			Mean (SD)	% with score ≥8
OAPs				
A. Self-management implies taking an active role in learning about one’s condition and in the shared decision-making process about one’s health and care pathway.	n.a	n.a	9.5 (0.6)	100
B. Self-efficacy (personal confidence to carry out an activity with the aim of achieving a desired outcome) has a positive effect on various aspects of living with IA.	n.a	n.a	9.6 (0.7)	100
C. Patient organisations often provide valuable self-management resources and collaboration between healthcare professionals (HCPs) and patient organisations will therefore benefit patients.	n.a	n.a	9.4 (1.0)	88
Recommendations				
R1. HCPs should encourage patients to become active partners of the team and make them aware of HCPs and patient organisations involved in all aspects of the care pathway.	5	D	9.5 (1.1)	87
R2. Patient education should be the start point and underpin all self-management interventions.	1A	A	9.5 (0.8)	93
R3. Self-management interventions that include problem solving and goal setting and, where relevant to the individual and available, cognitive behavioural therapy should be incorporated into routine clinical practice to support patients.	1A	A	9.1 (1.4)	93
R4. HCPs should actively promote physical activity at diagnosis and throughout the disease course.	1A	A	9.9 (0.3)	100
R5. Lifestyle advice based on evidence should be given to better manage common comorbidity and patients should be guided and encouraged by their healthcare team to adopt healthy behaviours.	5	D	9.6 (0.6)	100
R6. Better emotional well-being leads to better self-management; therefore, mental health needs to be assessed periodically and appropriate intervention should be made if necessary.	5	D	9.4 (1.3)	93
R7. HCPs should invite discussion with patients about work and signpost to sources of help where appropriate or where needed.	5	D	9.6 (0.5)	100
R8. Digital healthcare can help patients to self-manage and should be considered for inclusion in supported self-management where appropriate and available.	1B	A	9.3 (1.0)	93
R9. HCPs should make themselves aware of available resources to signpost patients to, as part of optimising and supporting self-management.	5	D	8.7 (1.2)	100

EULAR, European Alliance of Associations for Rheumatology; LoE, level of evidence (1–5; 1 indicating evidence from high-quality randomised clinical trial (RCT) data and 5 indicating evidence from expert opinion without explicit critical appraisal or based on physiology, bench research or ‘first principles’)^11^; n.a, not applicable; SoR, strength of recommendation (A–D; A indicating consistent level 1 studies (RCTs) and D indicating level 5 evidence or troublingly inconsistent or inconclusive studies of any level).

### Definition

The definition and concept of self-management varies widely in the published literature and the context in which it is used.^12^ The taskforce aligned mostly with the well-established definition of self-management provided by Barlow *et al* 13 whereby self-management is defined as ‘the ability of the individual to manage symptoms, treatment, lifestyle changes and psychosocial and cultural consequences of health conditions’. In this definition, two major components were highlighted: (1) self-management is aimed at achieving independence and (2) ideally, self-management should be supported by others, for example, HCPs, patient organisations and family. The taskforce proposed to emphasise the important contribution that patient organisations can make in supporting self-management for the purpose of this work and any future reference on the topic, something that has been largely overlooked and left out of most definitions to date.

### Overarching principles

The taskforce identified key themes considered to apply across all recommendations, formulated and agreed as three OAPs.

1. Self-management implies taking an active role in learning about one’s condition and in the shared decision-making process about one’s health and care pathway.
  Driven by the self-management definition above, it is important that patients take an active role in understanding their condition and engage in acquiring self-management skills and coping strategies, as well as in shared decision-making, as part of their care. Effective supported self-management encompasses the ability to monitor one’s condition and to put into action the cognitive, behavioural and emotional responses necessary to maintain a satisfactory quality of life.^14–17^ This way, a dynamic and continuous process of self-regulation is established. The importance of targeting and educating HCPs on self-management strategies and available resources, to ensure their ability to provide optimal support to patients, has been strongly emphasised.
2. Self-efficacy (personal confidence to carry out an activity with the aim of achieving a desired outcome) has a positive benefit on various aspects of living with IA.
  Good self-efficacy and coping skills benefit and reduce health and financial burden to the individual as well as the health service, benefitting society overall.^18 19^ Self-efficacy, supported by the existing literature, implies a process as well as an outcome^20^ since it is also an important outcome of self-management interventions.^1^
3. Patient organisations often provide valuable self-management resources and collaboration between HCPs and patient organisations may therefore benefit patients.
  There are numbers of best practice examples which include self-management resources in Europe, with important benefits for patients. Aside from practical advice and physical support, patient organisations can provide support with mental health issues, self-isolation and loneliness,^21^ which commonly feature in patients with IA. HCPs should take responsibility for addressing these issues in people living with IA and signpost to patient organisations. The taskforce acknowledges that variation exists both in healthcare delivery and the resources that patient organisations can offer. In some countries such as the UK, patient organisations invite HCPs to become medical advisors to the organisation and also provide free membership to all HCPs. Their medical advisors actively contribute educational articles for their magazines and to patient-related campaigns, educational activities and others. There is a close relationship that encourages cross-talk and collaboration that can be of huge benefit to patients.

### Recommendations

#### R1. HCPs should encourage patients to become active partners of the team and make them aware of HCPs and patient organisations involved in all aspects of the care pathway

For patients to take a more active role in their health, it is important that they are introduced to all members of the MDT involved in all aspects of their disease. Patient organisations can provide an invaluable source of information and resources to support patients. Yet, there seems to be a general lack of awareness of the self-management resources (and potential value) provided by many patient organisations (eg, in terms of patient education/disease knowledge, advocacy and other resources) and hence referral to these resources by HCPs. Some patients already engage in self-management and reach out to patient organisations for support. We acknowledge that patient organisations or at least well-developed patient organisations are not always available in many parts of Europe. Where available, patients should be signposted to relevant patient organisations in parallel with all other care and treatment they may be receiving.^22 23^ Where not available, we recommend using existing sources of information from the websites of other patient organisations and generally from trusted internet information sites, books and any other educational material that may be easily accessible online or via other routes.

#### R2. Patient education should be the start point and underpin all self-management interventions

Specific interactive education was among the most studied intervention across 19 randomised clinical trials (RCTs) based on the findings of the systematic review informing this taskforce (under submission). Self-management is considered a complex intervention as it contains many interacting techniques, thus making it difficult to identify the most effective components.^18^ Patient education is considered crucial, but not sufficient, in the context of self-management and is included in a majority of interventions.^24–30^ Patient education has been shown to improve treatment adherence, based on clinical trial evidence, although patient sample and follow-up time were both limited.^26^ The taskforce considers treatment adherence (and discussions addressing this) to be part of the patient education plan.^31^ Patient organisations reinforce the information and messaging about adherence and the impact of peer reinforcement around adherence is very powerful.

EULAR has produced recommendations for patient education for people with IA addressing when and by whom patient education should be offered, as well as modes and methods of delivery, theoretical frameworks, outcomes and evaluation.^32^ We advocate the use of these recommendations when it comes to patient education, recognising that patient education is an integral part of supported self-management for people with IA throughout the course of their disease.

#### R3. Self-management interventions that include problem solving and goal setting and, where relevant to the individual and available, cognitive behavioural therapy (CBT), should be incorporated into routine clinical practice to support patients

There are various self-management interventions. These include problem solving^27 29 30 33–39^ and goal setting,^27 29 30 33 34 37 39^ as well as cognitive behavioural therapy (CBT),^28 33 35 37–41^ supported by several SLRs and RCTs. The three interventions highlighted in this recommendation were therefore supported by strong evidence in their role in self-management. We advocate that they are promoted and provided where available and are relevant to patients, to enhance their ability to manage their disease confidently.^42^ CBT is a psychosocial intervention, often delivered by psychologists/psychotherapists, but also by some nurse specialists who have done a course in CBT and this further highlights the important role of the MDT. Referral to CBT can be initiated by any HCP involved in the care of the patient, if any doubt, in liaison with an expert delivering the intervention.

#### R4. HCPs should actively promote physical activity at diagnosis and throughout the disease course

Ample evidence from the existing literature supports the use of physical activity in IA and demonstrates its beneficial effect on several outcomes.^43–49^ Existing EULAR recommendations on physical activity^50^ emphasise its importance in disease management, based on proven effectiveness, feasibility and safety. Physical activity should thus form an integral part of standard patient care and be actively promoted and tailored to the individual’s circumstances, throughout their disease course. HCPs should be aware of the benefits of physical activity and advocate this as an important component of self-management. Any HCP should be able to promote the benefits of being physically active and take regular exercise and initiate a referral for physical therapy if deemed appropriate. If discussion is required with a physiotherapist or other physical exercise expert regarding the need and type of physical activity appropriate for an individual, then HCPs should know whom to approach for this. While there is a considerable amount of evidence for the beneficial effects of exercise, there is a general lack of emphasis on this aspect of care. Most interventions in regard to exercise relate to referral to a physiotherapist. However, the taskforce emphasises the importance and potential of exercise programmes and information provided by patient organisations and other community programmes, for example, classes which might include physical activities such as aquarobics, swimming, dancing, yoga and pilates.^51^

#### R5. Lifestyle advice based on evidence should be given to better manage common comorbidity and patients should be guided and encouraged by their healthcare team to adopt healthy behaviours

A number of modifiable lifestyle factors in IA can affect outcome.^52^ For example, the negative effects of smoking^53^ as well as high body mass index^54^ impact on inflammation and disease activity are now well established, as is the increased risk of cardiovascular disease.^55^ Lifestyle approaches should complement medical treatments, as also supported by a EULAR taskforce dedicated to providing recommendations on specific lifestyle interventions for the management of RMDs (currently ongoing). This taskforce considers such interventions to be a core part of self-management and advocates that patients receive support to adopt healthy behaviours including guidance on what constitutes a healthy, balanced diet, the benefits of exercise and quitting smoking, among others. Where specialised input is needed, for example, on nutrition, the input from dieticians should be sought where possible, acknowledging that dieticians are not always ‘standard’ members of the MDT so external support might be required. Such interventions are expected to have a positive impact on comorbidities and extra-articular manifestations, as well as the IA itself and should be accompanied by relevant investigations such as lipid profile testing, blood pressure monitoring and sleep hygiene.^4 6 56 57^ The addressing of comorbidities and initiation of relevant investigations may be undertaken by primary care physicians, rheumatologists or other HCPs such as nurses, involved in the patient’s care and as part, for example, of an annual review clinic. Some centres have their own pro formas for screening of comorbidities or lifestyle factors, for example, smoking, and these can be helpful as part of the screening process and facilitate the process for any member of the MDT.

#### R6. Better emotional well-being leads to better self-management; therefore, mental health needs to be assessed periodically and appropriate intervention be made if necessary

Poor mental health leads to worse outcomes in IA.^58–60^ CBT and other psychosocial interventions^43 61–64^ should be offered where available and tailored according to individual needs. Addressing mental health issues can help mitigate self-isolation and feelings of loneliness and can result in better self-management.^59 65^ Examples of questionnaires that could be used to measure patients’ emotional well-being feasibly in routine clinical practice include the mental health component of the SF36^66^ and the Patient Health Questionnaire (PHQ-9).^67^ The taskforce acknowledges that many patient organisations provide forums for networking and peer support programmes which can improve emotional well-being. Furthermore, we acknowledge that patients requiring more specialist assessment and support for mental health issues should be signposted as necessary, for example, to psychology and/or psychiatry.

#### R7. HCPs should invite discussion with patients about work and signpost to sources of help where appropriate or where needed

EULAR’s current strategy states that ‘by 2023, EULAR’s activities and related advocacy will have increased participation in work by people with RMDs’.^68^ The greatest proportion of people with IA are of working age at the time of diagnosis and work represents a major contributor to financial independence, self-esteem, purpose in life and overall quality of life.^2 69 70^ Therefore, it is crucial to the taskforce that HCPs address work-related aspects and signpost the patients to useful resources and support them to stay in work and maintain their independence.^71^ Occupational therapists and occupational health experts can provide helpful advice and resources in relation to the workplace.

#### R8. Digital healthcare can help patients to self-manage and should be considered for inclusion in supported self-management, where appropriate and available

Electronic patient records and other digital resources such as mobile health apps are becoming increasingly available in healthcare delivery.^72^ Mobile health technologies in particular can support self-management and allow people to take a more active role in their health.^72 73^ Patient-reported outcome domains as deemed relevant and important by patients could also be considered with digital healthcare. EULAR recommendations provide guidance on important aspects that should be considered for the development, evaluation and implementation of existing and new apps.^74^ The taskforce recommends referring to EULAR guidance on the above.

#### R9. HCPs should make themselves aware of available resources to signpost patients to, as part of optimising and supporting self-management

The taskforce highlighted the need for HCPs to be made aware of available resources for patients with IA, including those provided by patient organisations, to promote and support self-management interventions. At the same time, the taskforce recognised that just as there’s variation in healthcare resources, there is also variation in what patient organisations can offer.^75^

## Discussion

This EULAR taskforce has produced three OAPs relevant to nine agreed recommendations for the implementation of supported self-management strategies in patients with IA. OAPs and recommendations were met with strong consensus among experts in the task force.

The concept of self-management to some may imply needing to deal alone with a chronic condition.^76^ Receiving adequate support from a variety of sources is crucial.^77^ A key role of HCPs is also to enable access to and to signpost to supported self-management resources. Many HCPs will need to make themselves aware of how to most effectively provide and signpost to these different resources. The taskforce highlighted the importance of honesty and building trust as important elements for establishing open communication between patients and HCPs.^78–81^ Adequate time should be given to patients, as well as family and carers to discuss concerns and management options.^82 83^ Forward planning should be based on goal setting and what matters to patients, as supported by the existing literature.^27 29 30 33 34 37 39^ Furthermore, it was recognised that context, in other words, health system, culture or local resources, vary across settings and that nothing can be implemented without a clear familiarity and understanding of the local context. It is therefore important to understand and appreciate individual circumstances and social context when it comes to patient care, to maximise chances of implementing proposed care and supported self-management plans.^84 85^ For example, potential barriers to effective engagement with self-management could include poor health literacy and cultural or personal barriers, for example, for the latter, language barriers and low education. These should be identified where possible to maximise the support given to patients and to enhance their overall participation in self-management strategies. In some countries, patient organisations are particularly influential and with well-developed, active websites, support lines, educational material and some even with self-management programmes already established and made available to patients, families and carers. We encourage the use of social media such as Facebook, Twitter, websites and advertisements, for example, on national TV/radio to promote these resources.

Exploration of various definitions of self-management by the task force indicated that more holistic definitions of self-management reflecting the ‘individual’s ability to manage symptoms, treatment, physical and psychosocial consequences and lifestyle changes inherent in living with a chronic condition’ were more warmly received.^3^ The taskforce additionally highlighted the important contribution that patient organisations can make in supporting self-management, an aspect that has been largely left out of definitions to date. The latter is supported by additional sources of evidence informing this task force including direct communication with chief executives of patient organisations and best practice examples (available on request). However, the taskforce noted that the constitution of patient organisations varies considerably from large professional expert organisations led by paid chief executive officers and staff governed by boards of trustees to very small organisations which are primarily volunteer led. This means that the resources provided by patient organisations also vary.

Patient education has been identified as a crucial component that should underpin all self-management interventions. Effective patient education should be the responsibility of both the HCPs and the patients themselves. Patient education has been shown to improve treatment adherence,^26^ something that this taskforce recognises as an important part of patient education. Furthermore, patient outcomes including effective disease knowledge, healthcare management and self-efficacy have been shown to improve with patient education.^24–30^

The vision of the taskforce is that patient–HCP communication, the setting of meaningful and achievable goals and shared decision-making are seen as core components of self-management. This aligns well with EULAR’s current quality-of-care strategy that by 2023, EULAR will deliver pre-eminent comprehensive quality of care frameworks for the management of people with RMDs. One of the main quality-of-care objectives is to provide a ‘package’ that will enable greater uptake of the advice given in the recommendations, in other words emphasis on implementation aspects.^68^ In this regard and in relation to the nine recommendations, the taskforce recognised the importance of:

1. Raising awareness and educating HCPs on self-management strategies and available resources, to ensure ability to provide optimal support to patients.
2. Efforts to increase awareness and strengthen collaborations between patients, patient organisations and HCPs.
3. Signposting patients to good evidence-based information, also provided by many patient organisations.
4. Patient education as a crucial component of self-management, while acknowledging that being educated around various aspects of the disease does not necessarily imply implementation of meaningful changes.

It was particularly highlighted that training of HCPs, for example, on CBT, can improve their skills to deliver interventions and can be of great benefit to patients.^28 33 35 37–41^ The taskforce emphasised the need and importance of members of the MDT to be encouraged to work as a team towards implementation of the specific recommendations. Knowledge sharing should form a core part of these MDT meetings. Additionally, individual needs and variation in national health systems, availability of local resources and patient organisation offerings should be considered as part of the implementation. Finally, it is important to keep in mind that for self-management to be effective, the mode of delivery of various interventions should be considered in the setting of disease and severity, individual social circumstances and available resources. Referral to occupational health, occupational therapy patient organisations for resources related to work issues and other support should be considered where indicated and available.

With the recognition of all the above, unmet need has been identified and a research agenda has been proposed (box 1) for future work on the subject. An important focus has been the value of patient organisations and information and other resources they can provide to support people with IA, as well as the need to demonstrate and document the effectiveness of specific self-management interventions. It is particularly challenging for patient organisations to demonstrate the value of what they do, however, this does not remove the need for them to make real effort to demonstrate the impact of their resources. The taskforce identified, as part of the educational agenda, that there is scope for using best practice self-management programme examples to encourage and support other less-developed patient organisations and healthcare systems to work towards developing similar patient resources. Furthermore, in current clinical practice there is a strong emphasis on achieving clinical markers that are of importance to HCPs, for example, lowering of disease activity, whereas this taskforce is advocating that more focus should be given to goals that are more meaningful to the patients in the context of their everyday lives. In this respect, we recommend raising awareness among HCPs of the importance of the biopsychosocial determinants of health.

Box 1 **Research agenda**

Self-management in inflammatory arthritis (IA)—identified unmet need and suggested focus for future research.

1. To demonstrate the effectiveness of specific self-management interventions in IA and their impact on disease activity.
2. To study specific patient-reported outcome domains potentially affected by self-management including pain, fatigue, sleep, emotional and physical well-being, disability, quality of life and self-efficacy and explore a core outcome set.
3. To elucidate the cost-effectiveness of specific self-management interventions and programmes delivered.
4. To study the role of patient organisations and explore the impact of these organisations and the resources and support they provide for people with IA.
5. To investigate the impact of remotely delivered self-management interventions compared with face-to-face interventions.
6. To explore how the European Alliance of Associations for Rheumatology community could implement strategies to support and enable less established patient organisations to adapt best practice examples to suit their local circumstances.

## Conclusions

In conclusion, EULAR recommendations are now available for the implementation of self-management strategies in patients with IA. A dissemination strategy is currently underway to enhance the uptake of these recommendations, through national organisations, patient organisations and educational programmes.
