# Supplementary material for: 2021 EULAR recommendations for the implementation of self-management strategies in patients with inflammatory arthritis
Source: Ann Rheum Dis. 2021 May 7;80(10):1278–85. doi: 10.1136/annrheumdis-2021-220249 (PMC8458093; doi:10.1136/annrheumdis-2021-220249)
Supplement: Supplementary data [file annrheumdis-2021-220249supp001.pdf]

Supplementary File

Results of surveys conducted amongst EULAR PARE patient organisations and EMEUNET rheumatologists in Europe

Many patient organisations across Europe (and of course elsewhere in the world) work extremely hard to invest in and develop for their beneficiaries, an array of evidence-based resources including: national helplines; publications/information; peer support services; exercise programmes; self-management programmes; youth support programmes and activities; audio visual and digital material (eg. apps) to support education and patient self-management and empowerment. An impression has been gained however, that many health professionals primarily prioritise and value the information and resources which come from within the rheumatology team or hospital unit for patients, rather than regularly and consistently sign-posting their patients to the resources provided by the patient organisations active in their local region or country, which could support and add value to the information provided by the healthcare team.

Given the important role that Patient Organisations play for their beneficiaries and the part they will play in disseminating and promoting our recommendations amongst patient populations, we felt it was important to test this impression. As a consequence, our Taskforce conducted two short surveys amongst the CEOs of a number of patient organisations within EULAR PARE and a number of EUEUNET rheumatologists respectively, and asked them the following questions:

Figure 1

Patient Organisation questions

- **Please provide details of your top 3 resources for patients with IA in your country which support/educate/enable supported self-management.**
- **Are all/majority of HPRs in your country aware of these top 3 resources?**
- **Do HPRs refer patients to these resources?**
- **Do you promote these resources directly to HPRs? If so, how?**

EMEUNET rheumatologist questions

- **Are you aware of resources for patients with IA in your country which support/educate/enable supported self-management?**
- **Do you refer patients to these resources?**
- **Do you promote these resources e.g. patients, to HPRs? If so, how?**

Both the patient organisations and the clinicians were also asked if they would help to drive the adoption of this Taskforce's recommendations in their country when published, to which request, everyone agreed.

Patient organisations who responded were as follows:

- Switzerland – RheumaLiga Switzerland
- Cyprus - Cytanet
- Norway - Norwegian Association for Rheumatic Diseases
- Germany - Deutsche RheumaLiga
- Denmark - Gigtforeningen
- UK – responses from both National Rheumatoid Arthritis Society (NRAS) and National Axial Spondyloarthropathy Society (NASS)
- Slovakia - Slovak League against Rheumatism
- Romania - Romanian League

Responses to the EMEUNET survey to rheumatologists were received from across 13 European countries:

- Italy
- Portugal
- Austria
- Romania
- Netherlands
- Greece
- Sweden
- Belgium
- France
- Norway
- Denmark
- United Kingdom

It is important to state that patient organisations vary across Europe in terms of size, whether they have a paid CEO and paid staff or are run by volunteers as well as the capacity and extent of their resources. Having said that, there are many who do provide high quality resources which are evidence-based and comply with in-country rheumatology standards and guidelines. Equally important to state is that the EMEUNET clinicians surveyed may not be typical of the average consultant who is treating patients routinely in a district general hospital setting, as some may be more involved in research, and four out of the above list are on this Taskforce because they have an interest and expertise in self-management, and are therefore naturally more aware of and supportive of patient organisations' services available to patients in their area.

The overall 'take' seems to be that patient organisations, especially those more proactive and more established, have great resources and some of them also good connections with HCPs in promoting their materials.

The 'problem' seems to be originating primarily from HCPs, in that they may not actively promote/or in some cases, even believe in the impact these resources have on the patients.

However, it appears that greater effort is being made by some patient organisations to raise awareness of their resources than the efforts being made by numbers of individual HCPs or rheumatology societies to adopt or make use of such resources.

Widely promoting and advertising resources, with not just the patients as the key 'targets/stakeholders' but importantly, the HCPs so that they are made aware of the importance and potential impact of these resources for patients' well-being is key.

Top Resources Provided by Patient Organisations

Table 1

Patient education, (pain, fatigue, mental health, etc.)	Staying in employment & Social Benefits	Campaigning & Patient Advocacy
Helpline/guidebooks/other educational resources	Healthy Life Information & Advice	Patient videos, webinars, etc.
Peer Support Network/Support Groups	Physical Exercise information and programmes	Informative Self-management Courses

Key Emerging Themes - CEOs

1. Many HPRs are unaware of the key services and resources patient organisations offer.
2. Longer established and larger patient organisations offer a variety of resources and have better connections with HPRs
3. In spite of the above, HPRs do not always seem to promote PO resources to their patients

Detail

- It is easier in smaller communities for POs to individually approach HPRs, despite this, referral to POs remains low
- Despite excellent resources provide by POs in some countries, there seems to be a general lack of awareness/referral to these resources by HPRs.
- Some POs target their resources to specific disease areas or at early/established disease.
- Much effort is made by POs to market their resources both locally and at national congresses.
- There is a lack of understanding of the huge value to patients provided by some POs in terms of education, advocacy and supported self-management resources.

Key Emerging Themes – EMEUNET
1 - Resource Availability, summary

1. Many patient organisations provide useful resources, yet there exist multiple barriers to their access.
2. Despite the presence of patient organisations and a wealth of material and information in many countries, these resources do not seem to be fully and widely made available or used unless the POs do the promotion themselves.
3. Some organisations have established youth groups within them which are particularly active, also on social media.
4. Many patient organisation resources have information on self-management and other aspects of care, but access is variable (often patient initiated, but sometimes also by HPRs).
5. Efforts are taking place at a national level in some countries to provide recommendations for non-pharmacological and non-surgical interventions in RA.

Resource Availability – detail

- Access much easier in bigger cities.
- Financial support lacking; in some cities pharma industry has established private programmes
- Many of these resources are more available in or for research settings.
- Most self-management programs are hospital dependent, and most importantly staff availability dependent
- Youth groups/social media engagement seem to be more impactful.
- The majority or many of the resources seem to be for patients with inflammatory arthritis (IA).

Key Emerging Themes – EMEUNET
2 - Referral/Promotion of resources, summary

1. Where HPRs refer patients to existing resources, they promote them at least in informal conversations or some also through Social media (e.g. Twitter), making people more widely aware.
2. There appears to be heterogeneity in pro-activity of HPRs in promoting such resources; reasons include a lack of knowledge of the existence of these resources (a common problem) or clinic capacity.
3. Referring to self-management programmes is not always possible due to multiple barriers (incl. geographical issues, inclusion criteria).

Referral/Promotion of resources – detail

- HPRs (not clinicians) seem to report greater contact and closer relationship with local patient organizations.
- Patient information leaflets are often available and can be used in clinic.
- From those HPRs who are aware of PO resources, not all share this info/make their colleagues aware of this information (no internal promotion).
- An important problem seems to be limited numbers of rheumatologists and perhaps even more so, limited numbers of HPRs.
- Referral mainly considered for those patients who may need the 'extra-help' rather than 'everyone'.
- Low engagement of HPRs is a problem.
- Where this seems to work best, is in HPRs who already have good liaisons/communications with POs.

Key Emerging Themes – EMEUNET
3 – Implementation – Summary

1. There is general enthusiasm in driving recommendations at national level, helping implementation.
2. There is awareness that the long term efficacy data of some self-management programmes is variable, but that there is a great deal of research to show that patients who learn about their disease and self-manage well have better long term outcomes.

Implementation – detail

- The recommendations are expected to provide a frame to identify the unmet needs in various countries.
- General views support the communication of these recommendations through EULAR/EMEUNET to National Rheumatology societies.
- HCPs with a good understanding of this notion are keen to get the message out more widely to all.

Milestones to achieve

- There is a need to establish stronger links and build the relationship between HCPs and POs and the publication of these recommendations will provide opportunity to achieve that.
- POs need to be able to demonstrate the value of what they provide; so for them there needs to be a strong emphasis on building in suitable evaluation of their key services and resources.
- Widely promoting & advertising available resources not just to patients but also to HCPs so that they are made aware of the importance and potential impact of these resources for patients' well-being, is key. This would enhance awareness and implementation of our recommendations.

The Value of our Taskforce

Insights gained from the work of this Taskforce could open doors to much greater awareness across Europe of the value and resources available through patient organisations.

The provision of guidance through our Taskforce could be crucial in highlighting the importance and use of appropriate and currently available resources to effectively implement supported self-management in IA. A major part of the implementation of the recommendations from this Taskforce will involve HPRs in making themselves aware of what's available to them in their area and forging greater collaborations with POs, rather than thinking that most of the self-management resources need to be developed by HPRs. An important message also for Patient Organisations, as mentioned in 'milestones' above, will be to ensure that their resources are appropriately evaluated to demonstrate efficacy and value. The convenors of this Taskforce, Ailsa Bosworth and Elena Nikiphorou as well as its members, are passionate believers that when patients and health professionals work together, this powerful combination has a better chance of realising the outcomes that really matter to patients. In summary, these direct communications highlight the value of our work and the recommendations we have developed in providing guidance on the importance and use of appropriate resources for self-management in IA as part of a routine care pathway.

For further detail on any of the individual replies related to these surveys, please contact Ailsa Bosworth at NRAS. Also if anyone would like access to any of the best practice examples gathered as part of this work, please contact: Ailsa@nras.org.uk
